# Implementation of an Enhanced Prenatal Checklist to Increase Rates of Counseling of Prenatal Fetal Aneuploidy Testing

**DOI:** 10.7759/cureus.61654

**Published:** 2024-06-04

**Authors:** Elizabeth Cochrane, Sara Wetzler, Nicola Tavella, Whitney Lieb, Noel Strong

**Affiliations:** 1 Maternal-Fetal Medicine, Obstetrics and Gynecology, Icahn School of Medicine at Mount Sinai, New York, USA; 2 Obstetrics and Gynecology, Icahn School of Medicine at Mount Sinai, New York, USA

**Keywords:** prenatal genetic testing, fetal aneuploidy testing, evidence-based practices, implementation, amniocentesis, chorionic villus sampling, non-invasive prenatal testing, nipt, genetics counseling, fetal aneuploidy screening

## Abstract

Aim

This study aims to assess the effect of implementing an enhanced prenatal genetic checklist to guide the provider’s discussion on both screening and diagnostic options for fetal aneuploidy testing at the initial prenatal visit.

Methods

A retrospective quality improvement (QI) project was performed at a single, large, urban academic medical center. The implementation of this project was prospective; however, data was examined retrospectively after the QI initiative was implemented for three months. Patients were included if they were less than 24 weeks gestational age with a live intrauterine gestation at their initial obstetric (OB) visit. Patients less than 18 years old at the initial OB visit were excluded. The results were analyzed using the statistical software R. Chi-squared tests were used to examine proportional differences between the pre- and post-intervention groups with respect to demographic and clinical characteristics and documented genetic counseling discussions.

Results

A total of 416 patients were included in the final cohort. As measured by documentation, the rate of discussion of diagnostic prenatal genetic testing increased significantly from the pre-intervention proportion of 54% to the post-intervention proportion of 72% (p < 0.001). In the subgroup analysis of patients with advanced maternal age, the rate of discussion of diagnostic prenatal genetic testing increased significantly from the pre-intervention proportion of 53% to the post-intervention proportion of 83% (p = 0.003), and the rate of genetics counseling referrals made at the initial prenatal visit increased significantly from 4% pre-intervention to 38% post-intervention (p < 0.001).

Conclusions

The use of an enhanced prenatal genetic checklist led to increased discussion of diagnostic fetal aneuploidy testing and increased rates of referral to genetics counseling.

## Introduction

The American College of Obstetricians and Gynecologists (ACOG) recommends that all patients be offered both screening and diagnostic testing for fetal aneuploidy assessment [1]. In a multicenter anonymous survey, Peterson et al. found that no provider group universally offered diagnostic testing [2]. While noninvasive prenatal testing (NIPT) has high detection rates for the commonly screened for aneuploidies, it remains a screening test, and its positive predictive values are very dependent on the patient’s risk factors, particularly maternal age [3]. Diagnostic prenatal tests such as chorionic villus sampling (CVS) or amniocentesis have extremely high sensitivity and specificity (i.e., a karyotype obtained in this fashion is considered to have a 99% sensitivity and specificity) for all patients regardless of age and evaluate for more abnormalities compared to NIPT [3].

Early introduction and discussion of diagnostic testing is critical, as CVS is typically performed between 11 and 13 weeks gestation and amniocentesis is typically performed between 16 and 23 weeks gestation [1,3]. Upon review of our prenatal clinic’s rate of discussing both diagnostic and screening fetal genetic evaluation at the initial obstetric (OB) prenatal visit, we identified that screening tests were more readily discussed than diagnostic tests. As such, we identified a need to improve our providers’ practices regarding incorporating a discussion for both screening and diagnostic prenatal genetic evaluation, not just screening via NIPT.

This quality improvement (QI) project modified the preexisting prenatal genetic checklist used at our institution during prenatal care and educated the providers before modification. The prenatal genetic checklist is routinely used by providers at the initial OB prenatal care visit; therefore, it was anticipated that the enhanced prenatal genetic checklist would be routinely used in clinical practice.

The study’s main objective was to determine if the enhanced prenatal genetic checklist led to significantly increased discussion of prenatal diagnostic genetic testing, as reflected in the electronic medical record (EMR) documentation. A secondary objective was to determine whether genetic counseling referral rates and rates of discussion of diagnostic testing improved among patients of advanced maternal age (AMA; >35 years or older at delivery).

## Materials and methods

This retrospective QI project was performed at Icahn School of Medicine at Mount Sinai, New York, USA. The implementation of this project was prospective; however, data was examined retrospectively once the QI initiative concluded its three-month implementation period. Upon completion of the QI initiative, approval for retrospective analysis was obtained from the Institutional Review Board at the School of Medicine at our institution on May 14, 2024 under the study ID number 23-00662. This study is reported using the Revised Standards for Quality Improvement Reporting Excellence SQUIRE 2.0 [4].

Patients were included if they were less than 24 weeks gestation with a live intrauterine pregnancy at their initial OB visit. Patients less than 18 years old at the initial OB visit were excluded. Patients were identified in our EMR by retrospectively reviewing our clinic’s patient schedules for “New OB Reg/Provider” encounter types. Demographic characteristics that were collected included maternal age at delivery, self-reported race and ethnicity, insurance coverage, genetic carrier status (genetic carrier screening that was performed prior to the initial OB visit, either in a preconception setting or prior pregnancy), prior pregnancy information (history of congenital fetal anomaly or fetal chromosomal anomaly), and medical comorbidities.

Prior to this study, a prenatal genetic checklist was routinely used by providers to help guide their discussion in our clinic at initial OB visits. Given the widespread utility of this prenatal genetic checklist, the planned intervention was to modify the preexisting prenatal genetic checklist (Table 1) and maintain its widespread use. Regarding the implementation of the planned intervention, provider education and training were performed prior to implementation. Providers who perform initial OB visits were educated via a lecture format on the risks, benefits, and alternatives of both fetal aneuploidy screening and diagnostic testing. During that lecture, the providers were familiarized with the enhanced prenatal genetic checklist.

**Table 1:** Prenatal genetic checklist EMR template AFP, alpha-fetoprotein; AMA, advanced maternal age; CVS, chorionic villus sampling; EMR, electronic medical record; NIPT, noninvasive prenatal testing; NT, nuchal translucency

Preexisting prenatal genetic checklist (pre-intervention)	Enhanced prenatal genetic checklist (post-intervention)
( ) NT ultrasound	( ) NT ultrasound
( ) NIPT	( ) If AMA or other indication (i.e., family history, prior affected child, and consanguinity): refer to genetics counseling
( ) Maternal serum AFP	( ) Diagnostic testing (CVS/amniocentesis): discuss with all patients
	( ) If considering or desires diagnostic testing: refer to genetics counseling
	( ) NIPT: discuss with all patients
	( ) Maternal serum AFP

Six months of documentation from initial OB visits, three months pre-intervention, and three months post-intervention were reviewed. The post-intervention cohort began one month after provider training and the implementation of the enhanced prenatal genetic checklist. Patient charts were manually reviewed in the EMR. As no other prenatal genetic testing or education interventions occurred during this time, changes in the rates of discussion of screening and diagnostic testing and genetic counseling referrals were attributed to the implementation of the enhanced prenatal genetic checklist.

The following were reviewed regarding ethical considerations of this QI project: insurance coverage of genetics counseling referrals, a possible increase in appointment duration, and a possible increase in rates of diagnostic procedures. After further discussion, it was deemed that this intervention would not add significant time to a provider’s visit and that the genetic counseling offices accepted the same insurance that the prenatal clinic accepts. Study data were collected and managed using Research Electronic Data Capture (REDCap) tools supported by institutional funding from the Clinical and Translational Science Awards (CTSA) grant UL1TR004419 [5,6].

The statistical software R was used for all analyses. Chi-squared tests and Fisher’s exact tests were used to examine proportional differences between the pre-intervention and post-intervention groups with respect to demographic and clinical characteristics and documented genetic counseling discussions. A two-sided p-value of <0.05 was considered statistically significant.

## Results

**Figure 1:**
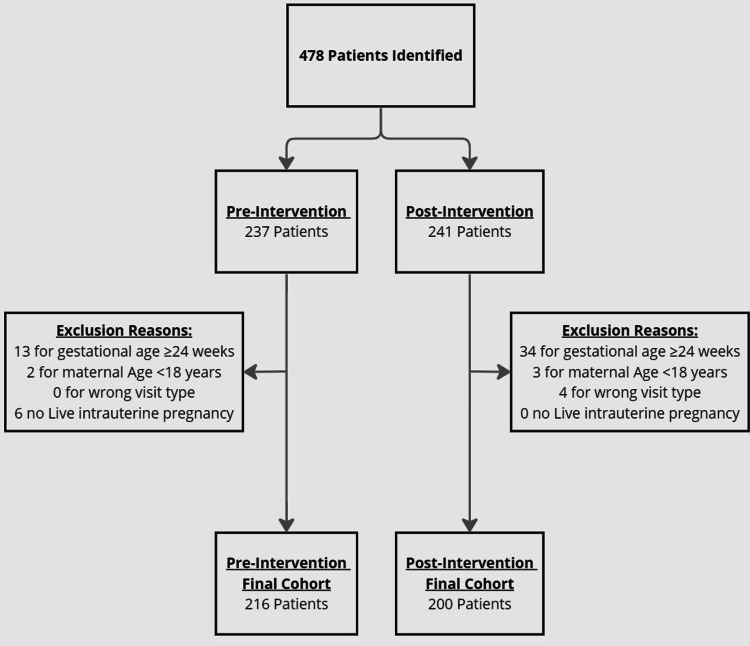
Flow diagram with exclusion criteria

A total of 478 records were identified; 237 patients received OB care pre-intervention and 241 received OB care post-intervention. Of all patients who presented to their initial OB visit, five were excluded due to age <18 years, 38 were excluded due to incomplete medical data, and 19 were excluded due to gestational age ≥24 weeks. There were 416 patients in the final cohort: 216 in the pre-implementation group and 200 in the post-implementation group (Figure 1).

Demographic information is displayed in Table 2. The post-intervention group had a significantly greater proportion of patients than the pre-intervention group with an unknown carrier status at the time of their initial OB appointment (78% vs. 65%, p = 0.01). The two groups did not differ significantly by any other demographic or clinical characteristic considered.

**Table 2:** Characteristics of the study sample and proportional differences between groups AMA, advanced maternal age

Characteristic	Total, n = 416, n (%)	Pre-intervention, n = 216, n (%)	Post-intervention, n = 200, n (%)	p-value
AMA at delivery				0.84
Yes	97 (23.3)	49 (22.7)	48 (24.0)	
Race and ethnicity				0.2
Asian	11 (2.6)	6 (2.8)	5 (2.5)	
Black/African American/Caribbean	134 (32.2)	74 (34.3)	60 (30.0)	
Hispanic/Latina	221 (53.1)	114 (52.8)	107 (53.5)	
White	19 (4.6)	12 (5.6)	7 (3.5)	
Other	28 (6.7)	9 (4.2)	19 (9.5)	
Insurance coverage				0.6
Public	395 (95.0)	203 (94.0)	192 (96.0)	
Private	19 (4.6)	12 (5.6)	7 (3.5)	
None	2 (0.5)	1 (0.5)	1 (0.5)	
Nulliparous				0.47
Yes	160 (38.5)	79 (36.6)	81 (40.5)	
Genetic carrier status				0.01
Yes	64 (15.4)	43 (19.9)	21 (10.5)	
No	56 (13.5)	32 (14.8)	24 (12.0)	
Unknown	296 (71.2)	141 (65.3)	155 (77.5)	
Prior pregnancy with a chromosomal fetal anomaly				0.59
Yes	9 (2.2)	6 (2.8)	3 (1.5)	
Prior pregnancy with a congenital fetal anomaly				0.89
Yes	11 (2.6)	5 (2.3)	6 (3.0)	
Pregestational diabetes mellitus				1
Yes	9 (2.2)	5 (2.3	4 (2.0)	
Chronic hypertension				0.07
Yes	17 (4.1)	13	4 (2.0)	

Table 3 displays proportional differences between groups regarding the discussion of screening and diagnostic testing. In the total sample, 89% of patients were counseled on basic genetic screening via NIPT, and 63% were counseled on diagnostic prenatal genetic testing, and the rate of discussion of genetics screening did not differ significantly pre- and post-intervention (p = 0.20). However, the rate of diagnostic prenatal genetic testing counseling increased significantly from the pre-intervention proportion of 54% to the post-intervention proportion of 72% (p < 0.001). The rate of genetics counseling referrals made at the initial prenatal visit increased significantly from 8% pre-intervention to 16% post-intervention (p = 0.03). The rate of documented discussion of carrier testing did not differ significantly between groups (p = 0.23).

**Table 3:** Proportional differences of genetic counseling documentation between groups NIPT, noninvasive prenatal testing; OB, obstetrical

Characteristic	Total, n = 416, n (%)	Pre-intervention, n = 216, n (%)	Post-intervention, n = 200, n (%)	p-value
NIPT discussion documented				0.2
Yes	370 (88.9)	188 (87.0)	182.0 (91.0)	
Diagnostic testing discussion documented				<0.001
Yes	260 (62.5)	117 (54.2)	143 (71.5)	
Genetics counseling referral placed at OB visit				0.03
Yes	50 (12.0)	18 (8.3)	32 (16.0)	
Carrier screening discussion documented				0.23
Yes	372 (89.4)	189 (87.5)	183 (91.5)	

Table 4 shows a subgroup analysis among patients who were AMA at their initial OB appointment. Among patients who were AMA, the rate of discussion of diagnostic prenatal genetic testing increased significantly from the pre-intervention proportion of 53% to the post-intervention proportion of 83% (p = 0.003). The rate of genetics counseling referrals made at the initial prenatal visit increased significantly from 4% pre-intervention to 38% post-intervention (p < 0.001). Rates of documented discussion of genetic screening and carrier testing did not differ significantly within this subgroup.

**Table 4:** Proportional differences of genetic counseling documentation between groups among AMA patients AMA, advanced maternal age; NIPT, noninvasive prenatal testing; OB, obstetrical

Characteristic	Total, n = 97, n (%)	Pre-intervention, n = 49, n (%)	Post-intervention, n = 48, n (%)	p-value
NIPT discussion documented				1
Yes	92 (94.8)	46 (93.9)	46 (95.8)	
Diagnostic testing discussion documented				0.003
Yes	66 (68.0)	26 (53.1)	40 (83.3)	
Genetics counseling referral placed at OB visit				<0.001
Yes	20 (20.6)	2 (4.1)	18 (37.5)	
Carrier screening discussion documented				0.17
Yes	88 (90.7)	42 (85.7)	46 (95.8)	

## Discussion

In 2020, ACOG recommended that providers discuss and offer genetic screening and diagnostic testing to all patients, not only those who are at increased risk for fetal aneuploidy [1]. Peterson et al. (2023) found that provider groups do not universally offer diagnostic testing, despite this ACOG recommendation [2]. This study evaluated the rate of discussions, as noted by EMR documentation, of genetic screening and diagnostic testing as well as the number of referrals for genetic counseling during a three-month period prior to implementation of the enhanced prenatal genetic template and a three-month period after implementation. We found a significant increase in rates of discussion of diagnostic prenatal testing and genetic counseling referrals after the implementation of our intervention. This finding was evident for the entire patient population as well as for a subgroup of patients who were AMA. Incidentally, we found a significant difference in unknown carrier status between the two cohorts; this is reflective of the care the patients received prior to the initial OB visit (i.e., prior pregnancy or preconception visit) and not the intervention. The rates of discussion of carrier screening were not significantly different between the pre- and post-implement cohorts.

Before the enhanced prenatal genetic checklist was introduced, many providers may not have offered, discussed, or adequately documented their discussion of prenatal genetic diagnostic testing at the first OB visit. Further, studies have demonstrated that OBGYN providers lack knowledge of genetic risks and their management, highlighting the need for updated practice guidelines regarding screening and diagnosis [7,8]. Including the enhanced prenatal genetic checklist in the EMR showed an improvement in the quality of patient care, both in fetal aneuploidy evaluation counseling and early referrals to genetic counseling. This aligns with other studies that demonstrated the success of checklists when used routinely in various settings, including surgery, inpatient hospitalizations, and high-reliability organizations (e.g., aviation, firefighting, and emergency departments) [9-14]. In obstetrics, checklists have been useful in improving outcomes for women receiving oxytocin, managing eclampsia, reducing the risk of morbidity and mortality for maternity patients, and preventing thromboemboli following a cesarean delivery [15-20]. Checklists are useful since they help physicians not forget to carry out certain tasks [15], i.e., discussing genetic screening and diagnostic testing during the prenatal visit, which is important for the provision of standardized and high-quality care.

Comprehensive genetic prenatal counseling is crucial for informed consent, and this should include a conversation regarding available evaluation options (i.e., diagnostic and screening evaluations), the risks and benefits of each test, and alternatives to testing [21-24]. Patients cannot be properly counseled on screening tests such as NIPT without discussing diagnostic tests such as CVS or amniocentesis. Furthermore, the nuances of prenatal genetic test results should be explained to patients prior to performing the test, as some patients may be familiar with the results from NIPT (including the risk of trisomy 21, 18, or 13), but they may be less familiar with the diagnostic testing capabilities available to them, such as comprehensive chromosomal aneuploidy assessment [25]. Patients should also comprehend the potential for uncertain results or incidental findings prior to the testing rather than afterward [26].

Importantly, the use of the enhanced prenatal genetic checklist at our institution demonstrated an improvement in discussions of prenatal fetal aneuploidy screening and diagnostic testing among patients who are AMA. The improvement among this subgroup highlights the effectiveness of including this template in the EMR, as providers may neglect to offer adequate genetic prenatal counseling, even among high-risk patients.

Strengths and limitations

The strengths of the study include a relatively large population at a high-volume institution in an urban setting, improved documentation, and the capability to extract data systematically from a comprehensive EMR that housed both the documentation of the prenatal visit as well as the referral to genetic counseling.

Given the retrospective nature of this study, a main limitation is that, while we could evaluate the quality of the documentation, we could not evaluate the quality of the discussion itself. Similarly, there was the possibility of providers falsely documenting a discussion about screening and diagnostic options when the counseling was not performed. It is also possible that before the template’s introduction, providers offered this counseling but failed to document it in the EMR. Another limitation of this study was that it was dependent on providers using the pre-customized checklist. If the provider had used their own checklist rather than the departmental checklist, they would not have seen the enhanced prenatal genetic checklist.

This study did not assess whether the patients attended the genetics counseling appointment that was scheduled, nor did it assess the rate at which screening and diagnostic tests were performed.

## Conclusions

Implementation of an enhanced prenatal genetic checklist increased rates of discussion of diagnostic fetal aneuploidy testing and referrals to genetics counseling. Our findings demonstrate the important role of providers in counseling and engaging patients in an informed discussion about prenatal genetic evaluation early in pregnancy. With this improvement in counseling, patients can make informed decisions regarding the prenatal genetic evaluation process, what it entails, and what the results mean. This study also demonstrates the value of templates in the EMR that can remind and encourage providers to engage in extensive and comprehensive counseling and discussions during the prenatal OB visit. Further research should evaluate the quality of counseling provided during fetal aneuploidy evaluation discussions, follow-up discussions regarding abnormal genetic testing results, and explore the rates of closed-loop referrals to a genetic counselor.
